# A Brief History of Mitochondrial Pathologies

**DOI:** 10.3390/ijms20225643

**Published:** 2019-11-12

**Authors:** Salvatore DiMauro

**Affiliations:** Department of Neurology, Columbia University Medical Center, New York, NY 10032, USA; sd12@cumc.columbia.edu

**Keywords:** mitochondrial pathologies, mtDNA mutations, mendelian and maternal inheritance

## Abstract

The history of “mitochondrial pathologies”, namely genetic pathologies affecting mitochondrial metabolism because of mutations in nuclear DNA-encoded genes for proteins active inside mitochondria or mutations in mitochondrial DNA-encoded genes, began in 1988. In that year, two different groups of researchers discovered, respectively, large-scale single deletions of mitochondrial DNA (mtDNA) in muscle biopsies from patients with “mitochondrial myopathies” and a point mutation in the mtDNA gene for subunit 4 of NADH dehydrogenase (*MTND4*), associated with maternally inherited Leber’s hereditary optic neuropathy (LHON). Henceforth, a novel conceptual “mitochondrial genetics”, separate from mendelian genetics, arose, based on three features of mtDNA: (1) polyplasmy; (2) maternal inheritance; and (3) mitotic segregation. Diagnosis of mtDNA-related diseases became possible through genetic analysis and experimental approaches involving histochemical staining of muscle or brain sections, single-fiber polymerase chain reaction (PCR) of mtDNA, and the creation of patient-derived “cybrid” (cytoplasmic hybrid) immortal fibroblast cell lines. The availability of the above-mentioned techniques along with the novel sensitivity of clinicians to such disorders led to the characterization of a constantly growing number of pathologies. Here is traced a brief historical perspective on the discovery of autonomous pathogenic mtDNA mutations and on the related mendelian pathology altering mtDNA integrity.

## 1. Introduction

Two great discoveries were made in 1988 by two famous researchers, Anita Harding at the Department of Clinical Neurology, Institute of Neurology, Queens Square, London, UK [1], and Doug Wallace at Emory University, Atlanta, GA [2]. The first discovery identified large-scale single deletions of mitochondrial DNA (mtDNA) in muscle biopsies from patients with “mitochondrial myopathies” while the second discovery identified a point mutation in the mtDNA gene for subunit 4 of NADH dehydrogenase complex (*MTND4*), associated with maternally inherited Leber’s hereditary optic neuropathy (LHON) in a large American pedigree.

These two discoveries launched a new hunger for human disorders associated with mutations in the tiny mtDNA molecule: In the same year, 1988, our large group of “mitochondriacs” at Columbia University, led by Bud Rowland, clarified the genetic etiology of Kearns–Sayre’s syndrome (KSS), the eponym of Anita Harding’s discovery [3].

**Figure 1:**
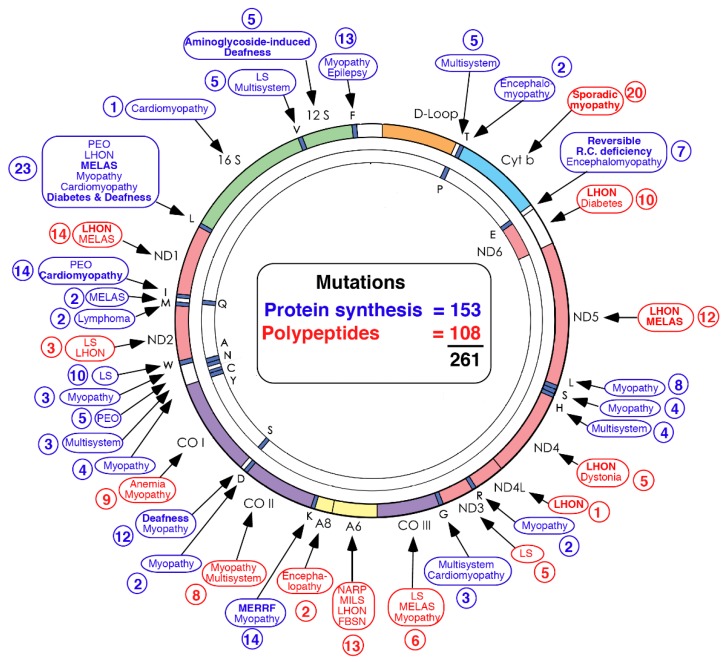
Morbidity map of mitochondrial DNA. Disorders caused by mutations in protein-coding genes are shown in red. Disorders caused by mutations in genes controlling protein synthesis are shown in blue.

Easy sequencing of mtDNA uncovered innumerable pathogenic mutations, reaching 281 in 2018, giving rise to a morbidity map of mtDNA that illustrates the tremendous phenotypic heterogeneity of mtDNA diseases (Figure 1).

This sensitized clinicians to a novel conceptual “mitochondrial genetics”, separate from mendelian genetics, based on three principles [4,5]:

(1) Polyplasmy, homoplasmy, heteroplasmy. Each cell contains hundreds or thousands of mitochondria and each mitochondrion harbors multiple copies of mtDNA (“polyplasmy”). Normal cells have identical copies of mtDNA (“homoplasmy”), but in patients with mutations in mtDNA we find a stochastic distribution of mutant and wild-type mtDNAs (“heteroplasmy”). A crucial corollary of heteroplasmy is that a critical number of mutant mtDNAs is needed for the respiratory chain to suffer and for disease to manifest (“threshold effect”). 

Mitochondria are ubiquitous organelles, present in all tissues (except mature erythrocytes), explaining why mtDNA diseases can affect any or all tissues, giving rise to the most heterogeneous clinical presentations. Multisystem disorders are typical examples of mtDNA diseases, including KSS, MELAS (mitochondrial encephalomyopathy, lactic acidosis, and stroke-like episodes), MERRF (myoclonic epilepsy and ragged-red fibers), NARP/MILS (neuropathy, ataxia, retinitis pigmentosa/maternally inherited Leigh’s syndrome).

However, there are rare cases in which individual tissues may be affected, for example skeletal muscle with myopathy due to mutations in the *cyt b* gene [6].

In this situation, we postulated that the mutation load of mutant *cyt b* was disproportionally high in skeletal muscle compared to other tissues, such as the mutation load surpassed the threshold effect. But typically mtDNA-related diseases are a heterogeneous group of clinical disorders. In rare instances, homoplasmic mtDNA mutations are known to cause diseases, such as LHON.

(2) Maternal inheritance. Following the evidence provided by Giles et al. [7], most pathogenic point mutations of mtDNA are transmitted maternally. An affected or unaffected mother carrying a mutant mtDNA can pass it on to all her children—boys and girls—and the disease can show, more or less severely, in all matrilinear members. Thus, it is important to collect family histories, including “soft signs” such as short stature, exercise intolerance, diabetes mellitus, or migrainous headache in maternal relatives. Family history clearly distinguishes maternally inherited mtDNA point mutations from diseases caused by large-scale single mtDNA deletions and a large survey of patients with KSS has shown the vast majority of sporadic patients [8]. 

A disturbing paper has recently been published [9], in which the authors question the dogma of mtDNA maternal inheritance. They convincingly showed variable heteroplasmy (24% to 75%) of paternal mtDNA in three unrelated multigenerational families, examined by high-depth whole mtDNA sequencing. The implication of pathogenic paternal mtDNA mutation transmission remains to be documented, because in the same study, maternal inheritance was demonstrated to remain absolutely prevalent and in all the assayed families paternal transmission provided a limited contribution to mtDNA. Also, the hypothetical usage of paternal mtDNA transmission to reduce the consequences of maternal pathogenic mutations is far beyond. In fact, it will require the full comprehension of the mechanisms leading to elimination of paternal mtDNA in developing embryos, likely related to a still unknown autosomal dominant gene, whose mutation might prevent the usual processes.

(3) Mitotic segregation is the third rule of mitochondrial genetics. As cells, heteroplasmic for mtDNA, divide, they pass on to next generation various mutation loads of mtDNA (wild-type and mutant) and, thus, both the genotype and the phenotype may vary in time.

## 2. Diagnosis of mtDNA-Related Diseases

Three techniques have contributed valuable clues to the diagnosis of mtDNA-related diseases.

The first technique was the histochemical overlap of cytochrome c oxidase (COX) and succinate dehydrogenase (SDH) stains, introduced by my friend Eduardo Bonilla. 

**Figure 2:**
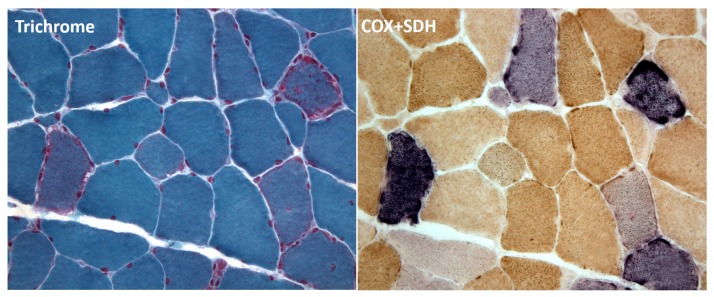
Cytochrome c oxidase (COX)/ succinate dehydrogenase (SDH) overlap staining in a muscle biopsy. This patient with myoclonic epilepsy and ragged-red fibers (MERRF) shows typical “ragged red fibers” (RRF) by trichrome at left. However, COX/SDH overlap staining (at right) shows COX-negative, “ragged-blue” fibers, mixed with COX-deficient bluish fibers.

COX deficiency is the hallmark of mtDNA diseases, with three enzymatically active mtDNA-encoded subunits (COXI–COXIII), while SDH (complex II) is considered a marker of mitochondrial overabundance as its four subunits are entirely encoded by nuclear DNA (nDNA). In patients with mitochondrial diseases, cross sections of frozen muscle biopsies clearly show scattered COX-negative or COX-deficient fibers, which are often overstained with SDH due to abnormal compensatory mitochondrial proliferation and identified as “ragged red fibers” (RRF) with the Gomori trichrome staining. The brown stain of COX prevails over the blue stain of SDH in normal fibers while COX-negative fibers show a bright blue color (“ragged-blue fibers”) and COX-deficient fibers will stain a milder bluish color [10] (Figure 2). This COX/SDH combined staining has proven diagnostically useful generally in muscle biopsies, but has been equally valuable in brain sections from patients with mtDNA-related encephalomyopathies [11].

**Figure 3:**
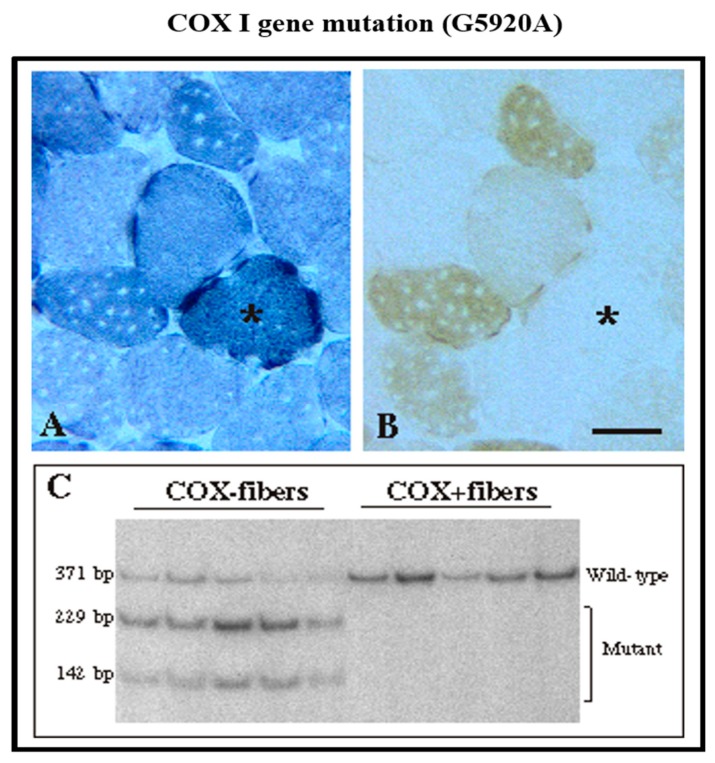
Evaluating the pathogenicity of a novel mtDNA mutation in single fibers by PCR. (**A**) Succinate dehydrogenase (SDH) staining; (**B**) Cytochrome c oxidase (COX) staining; (**C**) Analysis for a specific mtDNA mutation by PCR. In this young man with recurrent myoglobinuria, we identified a nonsense mutation in the *COXI* gene of mtDNA [13]. COX-negative (COX-) fibers are indicated by asterisks in A and B. The results of the PCR analysis in single fibers clearly show the high abundance of the mutation in COX- fibers and the lack of mutation in COX+ fibers.

The second—even more sensitive—technique was introduced by my great collaborator, Carlos Moraes [12], and based on PCR amplification of a novel mtDNA mutation. In particular, this method measures the relative percentages of wild-type and mutant mtDNA in COX-negative, COX-deficient, and normal-looking muscle fibers. These results give convincing clues to the pathogenicity of novel mtDNA variants. This required plucking from thick histochemical muscle sections COX-negative, COX-deficient, and normal-looking fibers and performing PCR on these tiny specimens (Figure 3). 

The third useful technique was the creation of the so-called “cybrid” (cytoplasmic hybrid) immortal fibroblast cell lines. It consists in transferring into rho-zero cells (deprived of their mtDNA) heteroplasmic mitochondria from cultured skin fibroblasts of patients carrying mtDNA mutations [14]. These heteroplasmic or homoplasmic cybrids provided hallmark models to evaluate biochemical consequences and functional threshold effects in MELAS, MERRF, and KSS. And, to say the least, hybrid cell lines were employed in various therapeutic strategies to test different approaches to treat mtDNA-related diseases.

The autonomy of mtDNA is wide enough to explain the multiple phenotypes in mtDNA-related disorders and provides valid rules of “mitochondrial genetics” to substantiate the features of those disorders, including multisystem or monosystem, maternal or sporadic inheritance, and progressive in time.

However, in 1989, my friends Massimo Zeviani and Stefano DiDonato found several Italian families with autosomal dominant or recessive mitochondrial progressive external ophthalmoplegia (PEO) and encephalomyopathy, in whose muscle biopsies they saw RRF and multiple deletions of mtDNA [15].

The partnership between the two genomes is not equal but overwhelmingly in favor of the nuclear genome, which controls mtDNA maintenance, replication and integrity. Pathogenic mutations in nuclear genes affecting mtDNA maintenance lead to two groups of mitochondrial diseases, characterized by mtDNA depletion or multiple mtDNA deletions. Their mendelian genetic origin shares in common phenotypical variegated expression with mtDNA-related diseases, due to the “threshold effect” of mtDNA damage.

The concept of mtDNA depletion was introduced in 1991 by Carlos Moraes, showing complete or partial mtDNA reduction in different tissues from severely affected infants [16]. At the same time, Eduardo Bonilla devised an immunochemical stain for mtDNA in contrast to bright nDNA to be applied in muscle biopsies [17].

Multiple nuclear genes are involved in controlling mitochondrial nucleic acid biosynthesis and the intramitochondrial pool of deoxynucleoside triphosphates (dTNPs), the building blocks of DNA.

Mutations in the *DGUOK* gene encoding deoxyguanosine kinase were described in Israel, causing a severe hepatocerebral syndrome with neonatal liver failure, nystagmus, and hypotonia [18].

Mutations in the *TK2* gene encoding thymidine kinase 2 are among the most common causes of mitochondrial depletion syndrome, with about 200 patients that have been reported by two groups, Hirano’s and Wang’s [19,20]. Most of the patients with *TK2* mutations have an infantile onset form, presenting at or soon after birth with generalized weakness, respiratory insufficiency, and death at 1 to 3 years of age. Severe skeletal muscle mtDNA depletion is commonly observed.

Lately, Michio Hirano and Caterina Garone have developed a life-saving therapeutic approach for these infants. The therapy provided oral deoxynucleosides (the substrates of the TK2 enzyme) and showed efficacy in ameliorating mtDNA depletion and increased lifespan in mice [21,22]. Up to today, this treatment has been used in 16 patients worldwide under compassionate use, the exiting results obtained in this cohort will lead to a forthcoming clinical trial. 

Mutations in the *SUCLA2* gene encoding ADP-forming succinyl-CoA synthase usually cause severe infantile encephalomyopathies [23] but they may present in childhood with psychomotor delay, deafness, myopathy, ataxia, and chorea [24]. Mutations in the *SUCLG1* gene encoding succinyl-CoA ligase have been associated with infantile hepatocerebral LS-like syndrome [25].

Mutations in the *RRM2B* gene encoding p53-inducible ribonucleoside reductase subunit 2B caused severe infantile disease, with hypotonia, tubulopathy, seizures, respiratory distress, diarrhea, and lactic acidosis [26] but application of whole exome sequencing (WES) has revealed unusual late-onset cases with PEO [27] or KSS [28], and a case with mitochondrial neurogastrointestinal encephalomyopathy (MNGIE syndrome) [29].

Mutations in the *TYMP* gene encoding thymidine phosphorylase (TP), identified by Michio Hirano [30], were associated with the severe syndrome of MNGIE. The syndrome usually affects young adults with intestinal problems (increased borborygmi, intestinal pseudoobstruction, diarrhea), PEO, severe peripheral neuropathy (axonal or demyelinating), and leukoencephalopathy. The disease course is severe, evolving in pronounced cachexia and early death. The pathogenesis of MNGIE is attributed to excessive accumulation of two toxic compounds, thymidine and deoxyuridine, causing mtDNA alterations (depletion, multiple deletions, and site-specific point mutations, isolated or in combination) [31].

Michio Hirano introduced a useful, if risky, therapy for patients with devastating MNGIE syndrome. It consists in allogeneic hematopoietic stem cell transplant (AHSCT, i.e., bone marrow transplantation), which showed in an international clinical trial to be able to detoxify the body from thymidine and deoxyuridine, alleviating neuropathy, and increasing body weight [32].

Massimo Zeviani’s group [33] discovered mutations in the *MPV17* gene encoding a protein in the inner mitochondrial membrane (IMM) of uncertain significance, causing a severe infantile hepatocerebral syndrome. To our recognition, we attributed *MPV17* mutations to the Navajo neurohepatopathy (NNH), an endemic disease in the Southwestern US, characterized by childhood neuropathy, acral mutilation, corneal scarring or ulceration, liver failure, and CNS demyelination [34].

Impairing mtDNA replication may cause severe mtDNA depletion, exemplified by Alpers–Huttenlocher syndrome (AHS) due to mutations in the *POLG* gene encoding polymerase gamma [35]. A triad of refractory seizures, psychomotor regression, and liver disease in children characterizes AHS. Patients with AHS do not survive long and are particularly vulnerable to administration of valproic acid (VPA) therapy.

However, innumerable mutations throughout the *POLG* gene—even a few heterozygous—cause a variety of phenotypes, including PEO-plus, sensory ataxic neuropathy, dysarthria, and ophthalmoparesis (SANDO), mitochondrial recessive ataxia syndrome (MIRAS), and PD, all reviewed by Dr. William Copeland [36].

Multiple mtDNA deletions were first attributed to mendelian genetics by Massimo Zeviani and Stefano DiDonato [15] and a woman that was described by Suomalainen, manifesting PEO and psychiatric disorder, carried multiple deletions of mtDNA in her muscle, brain, and liver [37]. In short sequence, multiple mtDNA deletions were related to the gene *ANT1* (ADP-ATP translocase) that was identified mutant in a Finnish family with autosomal dominant PEO [38]. Only few cases of autosomal dominant PEO have been recognized as caused by heterozygous mutations of the renamed *SLC25A4* gene, which encodes the ADP-ATP translocase. However, the relevance of nuclear genes encoding mitochondrial carriers (MC) has been recently increased because of the growing number of mutations discovered in MC genes that lead to MC-associated diseases. Such number will likely increase further, particularly because the role of more than 20 human genes of the *SLC25* family remains to be identified [39].

Mutations in the gene *TWNK* (or *PEO*) encoding the helicase Twinkle have been widely reported in families with autosomal dominant PEO [40] plus cardiomyopathy [41]. A frequent presentation of recessive *TWNK* mutations is infantile onset spinocerebellar ataxia (IOSCA) [42,43].

Mutations in the *TK2* gene show—beside infantile-onset myopathy—later-onset presentations with multiple mtDNA deletions in skeletal muscle [19,44]. A case of my friend Darryl De Vivo was puzzling for typical SMA (Spinal Muscular Atrophy) and mitochondrial myopathy, showing childhood mtDNA depletion and multiple deletions encephalomyopathy [45]. There were several adults with PEO-plus and both childhood- and late-onset patients with *TK2* gene mutations, who responded to oral deoxynucleoside therapy [22].

An unexpected adult with PEO-plus was affected with *MPV17* gene mutations [46] and adult-onset autosomal dominant or recessive PEO-plus was reported in patients with mutations in the *RRM2B* gene [27,28,29], and in patients with *DGUOK* gene mutations [47].

Finally, mutations in the *POLG2* gene, encoding the accessory dimeric subunit POLG2, enhancing binding to DNA, cause adult-onset dominant PEO [48] although we have recently found a homozygous *POLG2* gene mutation causing fatal infantile hepatic failure with mtDNA depletion [49] (Table 1).

Recent evidences also support a role for mtDNA damage and the related oxidative stress in the pathogenesis of atherosclerosis and vascular diseases. In fact, mtDNA oxidative damage is present at early stages of atherosclerosis and might promote the onset/progression of the disease because of its effects on mitochondrial and whole cell metabolism [50]. Also, the onset of vascular diseases appears related to the presence of genomic and mtDNA damage [51] and this is a further indication of the relevance that mtDNA alterations, even when not involving sequence mutations, might have for multifactorial diseases as many among the age-related diseases. More recently, studies involving sequencing of mtDNA regions from blood leukocytes of a large cohort of subjects have demonstrated the positive association of five specific mtDNA mutations with the incidence of carotid atherosclerosis suggesting that such mutations might be used to assess the predisposition to the disease [52]. Some mtDNA mutations from the most updated list of mutations potentially involved in atherosclerosis’ pathogenetic mechanisms [53] are presently under experimental test to verify their effective role in the disease’s onset. In fact, the creation of cybrids carrying different heteroplasmic levels of two mtDNA mutations, one associated with the presence and the other with the absence of atherosclerosis [54], will allow to thoroughly investigate the occurrence and the development of atherosclerosis in a quite simple cell model. This is the ultimate chronological step, but certainly not the last one along the pathway leading to the progressive understanding of the relevance of mtDNA mutations for monogenic as well as polygenic diseases.

## 3. Conclusions

This is my brief historical perspective on the discovery of autonomous pathogenic mtDNA mutations and of the related mendelian pathologies altering mtDNA integrity.

From the initial reports dating back to 1988, a long path has been followed with unexpected discoveries that opened an avenue leading to the always more detailed description of the newly identified mitochondrial pathologies as well as to a more comprehensive understanding of the crucial role of mitochondria inside cells, able also to send retrograde stress signals, which impinge on expression of nuclear genome and whole cell responses.

## Figures and Tables

**Table 1:** Intergenomic defects. Summary of multiple mendelian genetic defects affecting mtDNA integrity, causing—in various combinations—mtDNA depletion and mtDNA multiple deletions. Acronyms are explained in the text.

Gene	mtDNA Depletion	mtDNA Multiple Deletions
*DGUOK*	Hepato-cerebral syndrome	Adult-onset PEO
*TK2*	Lethal infantile myopathy	Later-onset myopathies
*SUCLA2*	Infantile encephalomyopathy	Later-onset movement disorder
*SUCLG1*	Hepato-cerebral syndrome	
*RRM2B*	Infantile encephalomyopathy	Adult-onset PEO; KSS; MNGIE
*TYMP*	MNGIE	
*MPV17*	Hepato-cerebral syndrome; NNH	Adult-onset PEO
*POLG*	Alpers–Huttenlocher syndrome	Adult-onset PEO; SANDO; MIRAS; PD
*POLG2*	Fatal infantile liver failure	Adult-onset PEO
*TWNK*	-	AD PEO; IOSCA
*ANT1*	-	Adult-onset PEO, dementia
*OPA1*	DOA	Adult PEO-plus

## Abbreviations

mtDNA	mitochondrial DNA
*Cytb*	Cytochrome b gene
COX	cytochrome c oxidase
SDH	succinate dehydrogenase
IMM	inner mitochondrial membrane
PD	Parkinson’s Disease
